# Hypertension and Stroke as Mediators of Air Pollution Exposure and Incident Dementia

**DOI:** 10.1001/jamanetworkopen.2023.33470

**Published:** 2023-09-20

**Authors:** Boya Zhang, Kenneth M. Langa, Jennifer Weuve, Jennifer D’Souza, Adam Szpiro, Jessica Faul, Carlos Mendes de Leon, Joel D. Kaufman, Lynda Lisabeth, Richard A. Hirth, Sara D. Adar

**Affiliations:** 1 Department of Epidemiology, University of Michigan School of Public Health, Ann Arbor; 2 Institute for Social Research, University of Michigan, Ann Arbor; 3 University of Michigan Medical School, Ann Arbor; 4 Institute for Healthcare Policy and Innovation, University of Michigan, Ann Arbor; 5 Veterans Affairs Center for Clinical Management Research, Ann Arbor, Michigan; 6 Department of Epidemiology, Boston University School of Public Health, Boston, Massachusetts; 7 Department of Biostatistics, University of Washington, Seattle; 8 Department of Oncology, Georgetown University, Washington, DC; 9 Department of Epidemiology, University of Washington, Seattle; 10 Department of Environmental and Occupational Health Sciences, University of Washington, Seattle; 11 Department of Medicine, University of Washington, Seattle; 12 Department of Health Management and Policy, University of Michigan School of Public Health, Ann Arbor; 13 Department of Internal Medicine, University of Michigan, Ann Arbor

## Abstract

**Importance:**

Fine particulate matter air pollution (PM_2.5_) has been consistently associated with cardiovascular disease, which, in turn, is associated with an increased risk of dementia. As such, vascular dysfunction might be a mechanism by which PM_2.5_ mediates dementia risk, yet few prior epidemiological studies have examined this potential mechanism.

**Objective:**

To investigate whether hypertension and stroke serve as mediators and modifiers of the association of PM_2.5_ with incident dementia.

**Design, Setting, and Participants:**

As part of the Environmental Predictors of Cognitive Health and Aging (EPOCH) Project, this cohort study used biennial survey data collected between 1998 and 2016 from respondents of the Health and Retirement Study (HRS), a nationally representative, population-based, cohort in the US. Eligible participants were those over 50 years of age who were free of dementia at baseline and had complete exposure, mediator, outcome, and demographic data from the HRS. Data analysis was conducted from August to November 2022.

**Exposures:**

Exposure to PM_2.5_, calculated for the 10 years preceding each person’s baseline examination according to residential histories and spatiotemporal models.

**Main Outcomes and Measures:**

Incident dementia was identified using a validated algorithm based on cognitive testing and informant reports. The 4-way decomposition causal mediation analysis method was used to quantify the degree to which hypertension and stroke mediated or modified the association of PM_2.5_ with incident dementia after adjustment for individual-level and area-level covariates.

**Results:**

Among 27 857 participants (mean [SD] age at baseline, 61 [10] years; 15 747 female participants [56.5%]; 19 249 non-Hispanic White participants [69.1%]), 4105 (14.7%) developed dementia during the follow-up period (mean [SD], 10.2 [5.6] years). Among participants with dementia, 2204 (53.7%) had a history of hypertension at baseline and 386 (9.4%) received a diagnosis of hypertension during the follow up. A total of 378 participants (9.2%) had a history of stroke at baseline and 673 (16.4%) developed stroke over the follow-up period. The IQR of baseline PM_2.5_ concentrations was 10.9 to 14.9 μg/m^3^. In fully adjusted models, higher levels of PM_2.5_ (per IQR) were not associated with increased risk of incident dementia (HR, 1.04; 95% CI, 0.98 to 1.11). Although there were positive associations of prevalent stroke (HR, 1.67; 95% CI, 1.48 to 1.88) and hypertension (HR, 1.15; 95% CI, 1.08 to 1.23) with incident dementia compared with those free of stroke and hypertension during follow-up, there was no statistically significant association of PM_2.5 _with stroke (odds ratio per IQR increment in PM_2.5_, 1.08; 95%CI, 0.91 to 1.29) and no evidence of an association of PM_2.5 _with hypertension (odds ratio per IQR increment in PM2.5, 0.99; 95%CI, 0.92 to 1.07). Concordantly, there was no evidence that hypertension or stroke acted as mediators or modifiers of the association of PM_2.5_ with incident dementia. Although the nonmediated interaction between PM_2.5_ and hypertension accounted for 39.2% of the total excess association (95% CI, −138.5% to 216.9%), the findings were not statistically significant.

**Conclusions and Relevance:**

These findings suggest that although hypertension may enhance the susceptibility of individuals to air pollution, hypertension and stroke do not significantly mediate or modify the association of PM_2.5_ with dementia, indicating the need to investigate other pathways and potential mediators of risk.

## Key Points

**Question:**

Does hypertension or stroke act as a mediator or modifier of the association of exposure to particulate matter air pollution (PM_2.5_) with incident dementia?

**Findings:**

In this cohort study of 27 857 individuals in the US, there was no evidence that hypertension or stroke acted as mediators or modifiers of the association of PM_2.5_ with incident dementia.

**Meaning:**

These findings suggest that the association of PM_2.5_ with dementia is not mediated or modified by hypertension or stroke, indicating the need to investigate other mediators and pathways.

## Teaser

This cohort study examines whether hypertension and stroke mediate the association of particulate matter air pollution with incident dementia.

## Introduction

Dementia is characterized by the loss of cognitive abilities severe enough to interfere with independent function in daily life.^1^ Because dementia is a major cause of disability and dependency among older adults,^2^ prevention efforts are of great importance, especially given that there is not yet a cure. Increasing evidence has identified fine particulate matter (PM_2.5_) air pollution as a novel, modifiable factor associated with risk of dementia,^3^ with associations reported in studies from Asia,^4^ Europe,^5,6,7^ and North America.^8,9,10,11,12^

Although the exact mechanisms underlying these associations remain unknown, PM_2.5_ may lead to activation of microglia, neuroinflammation, and dysfunction of the cerebrovasculature and nonneuronal neighboring cells, which may cause cerebral ischemia, hemorrhage, or atrophy. This process may occur by direct entry of PM_2.5_ into the brain through the olfactory nerves or via translocated particles that have entered the brain through the blood-brain barrier. PM_2.5_ could also indirectly affect the brain via activation of a localized inflammatory response in the cardiovascular system, triggering the release of additional inflammatory factors (eg, cytokines) into the circulation, which may lead to systemic inflammation or microglia activation and, ultimately, contribute to brain inflammation.^13,14,15^ Finally, PM_2.5_ may also impact the brain through the instigation of autonomic nervous system imbalance.^16^

Given the association of PM_2.5_ with vascular conditions,^17,18,19^ as well as the observed associations of vascular conditions with dementia,^20,21^ vascular dysfunction may act as a mediator of the association of PM_2.5_ with dementia. Via these pathways, as well as the shared mechanisms of inflammation and oxidative stress in the pathways from PM_2.5_ and vascular conditions to accelerate cognitive decline,^6,21^ it is possible that pollution exacerbates the impact of vascular disease on cognitive decline even if the vascular disease was not the result of air pollution exposure. To the best of our knowledge, however, only 2 studies^6,22^ have formally examined the role of cardiovascular disease (CVD) as a mediator in the association of air pollution with incident dementia. Both studies^22^ found some evidence of a mediated association through CVD, and 1 of the studies^6^ identified stroke as the most important mediator of these associations. To our knowledge, no study has yet evaluated vascular conditions as mediators and modifiers of these associations simultaneously or has evaluated these mechanisms in populations from the US.

In this study, we aimed to examine the roles of stroke and hypertension (a factor associated with risk of stroke^23^) as mediators in the association of long-term exposure to PM_2.5_ with dementia. We used a causal mediation approach to decompose the total association of PM_2.5_ with incident dementia into 4 components: the association related to (1) neither mediation nor interaction, (2) interaction only, (3) mediation only, and (4) both mediation and interaction.

## Methods

### Study Population

This cohort study followed the Strengthening the Reporting of Observational Studies in Epidemiology (STROBE) reporting guideline. All study activities, including data collection from the Health Retirement Study (HRS) and linkages of the HRS to environmental measures through the Environmental Predictors of Cognitive Health and Aging Project (EPOCH), were approved by the University of Michigan institutional review board. Written informed consent was obtained from all study participants by the HRS. The HRS is a nationally representative open cohort of older adults in the US. Since 1992, respondents have been interviewed biennially about their demographics, health, health behaviors, and residential histories until death or loss to follow-up.^24,25^ For participants who were unable or unwilling to be interviewed, a proxy would provide the answer to the survey questions. The sample for our analyses consisted of all participants over 50 years of age without dementia at baseline and at least 1 follow-up interview between 1998 and 2016. Participants with missing data on exposure, potential mediators, or key covariates were excluded (eFigure in Supplement 1).

### Air Pollution Assessment

As part of the EPOCH project, we estimated mean PM_2.5_ concentrations for each participant on the basis of their residential addresses during the 10 years prior to baseline as a marker of long-term exposure before disease development. For simplicity and concurrence with federal air pollution regulations, we focused our main analysis on total PM_2.5_. However, because we previously observed associations of incident dementia with PM_2.5_ from agriculture and open fires in this cohort,^26^ we also evaluated PM_2.5_ from these 2 sources in secondary analyses.

To estimate total PM_2.5_, we used spatiotemporal estimation models that have been described in detail elsewhere.^27,28^ Briefly, these models incorporate (1) measurements from the US Environmental Protection Agency regulatory networks and several research studies; (2) more than 300 geographic covariates characterizing nearby transportation, land cover and use, population density, emission sources, and vegetation; and (3) spatial and temporal correlations.^28^ We generated estimates for the HRS cohort between 1990 and 2016.

In secondary analyses, we characterized PM_2.5_ from agriculture and open fires by multiplying total PM_2.5_ with spatially varying fractional contributions of PM_2.5_ attributable to each source.^29^ McDuffie et al^29^ estimated these source fractions at a resolution of 0.5° × 0.625° by serially running an atmospheric chemistry-transport model (GEOS-Chem) with all sources but 1 to isolate the unique contribution of that source to the total PM_2.5_ mixture.

### Dementia Classification

HRS has collected cognitive function measures biennially since 1998. For self-respondents, this assessment included immediate and delayed word recall, the Serial 7s test, and counting backward. For proxy respondents, there were questions related to the participant’s memory, instrumental activities of daily living limitations, and interviewer assessment of the participant’s cognitive impairment. We then used the Langa-Weir classification algorithm^30^ to identify self-respondents with cognitive scores of 6 or fewer points of 27 points or proxy respondents with cognitive scores of 6 or more points of 11 points as having dementia.

### Vascular Conditions Assessment

We identified individuals as having hypertension or stroke if they or their proxies reported a history of doctor diagnosis for each condition during follow-up. We included both present or past vascular diseases because restriction to those free of hypertension or stroke could have introduced selection (collider) bias if long-term exposure were associated with vascular conditions before baseline and there were unmeasured common causes of earlier vascular conditions and dementia. In addition, using prevalence data also provided more statistical power.

### Statistical Analyses

**Figure 1.**
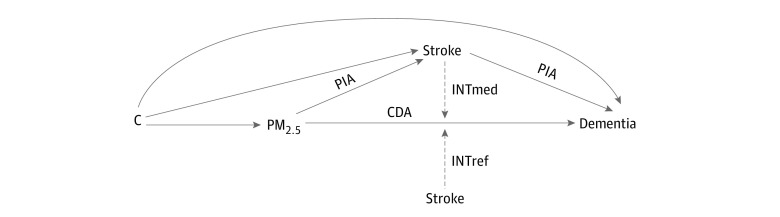
Conceptual Model for the Analysis of Stroke or Hypertension Mediating the Association of PM_2.5_ With Dementia Association by Vascular Conditions The figure shows how hypertension or stroke (stroke used as the example) could serve as mediators of the association of PM_2.5_ with dementia. *C* is the variable representing potential exposure-mediator, exposure-outcome, and mediator-outcome confounders. CDA indicates controlled direct association; INTref, reference interaction; INTmed, mediated interaction; and PIA, pure indirect association.

**Figure 2.**
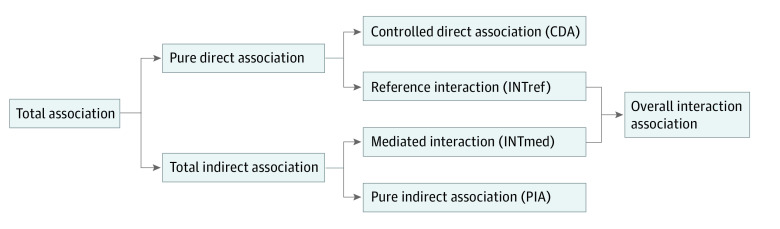
Illustration of the 4-Way Decomposition of Total Association The controlled direct association is due to neither mediation nor interaction. The reference interaction is only due to interaction. The mediated interaction is due to both mediation and interaction. The pure indirect association is only due to mediation.

Within the framework illustrated in Figure 1 and Figure 2, we decomposed the total association of PM_2.5_ with incident dementia into a controlled direct association (CDA), reference interaction (INTref), pure indirect association (PIA), and mediated interaction (INTmed).^31^ The CDA is due to neither mediation nor interaction and represents the direct association of PM_2.5_ with incident dementia when a person has never had a stroke or hypertension through the end of follow-up. The INTref is only due to interaction, representing that the association of PM_2.5_ with incident dementia is modified by hypertension or stroke status but hypertension or stroke is not due to the association with PM_2.5_. The PIA is only due to mediation, representing the association of hypertension and stroke with incident dementia where hypertension and stroke is first caused by PM_2.5_. INTmed is due to both mediation and interaction, representing that the association of PM_2.5_ with incident dementia is modified by hypertension and stroke and hypertension and stroke status is due to the association with PM_2.5_. The total indirect association is the sum of the INTmed and the PIA, and the overall interaction association is the sum of INTmed and INTref.

To begin our exploration of these mechanistic pathways, we first evaluated the association of PM_2.5_ with the mediators using logistic regression models with prevalent hypertension or stroke as our outcome. We then tested whether individuals with prevalent hypertension or stroke were more likely to experience incident dementia using Cox proportional hazard models. Next, we estimated the CDA, INTref, INTmed, and PIA separately using the 4-way decomposition causal mediation methods.^31^ Given the stratified multistage sample design of HRS, we applied person-level sampling weights and included geographic stratification and clustering in all models to obtain the correct SEs for the estimated associations.^32^

To assess the direct and indirect associations of PM_2.5_ with incident dementia, we first used Cox proportional hazards model following the general form in Equation 1 (using stroke as an example), where PM_2.5_ and stroke correspond to the main associations of time with dementia (*T*), PM_2.5_ × stroke is the interaction between PM_2.5_ and stroke status, and *C* is a matrix of potential exposure-mediator, exposure-outcome, and mediator-outcome confounders at baseline:

λ(*T*|PM_2.5_, stroke, *C*) = λ_0_exp(θ_1_PM_2.5_ + θ_2_stroke + *^c^*θ_3_PM_2.5_ × stroke + θ′*C*) (1).

In *C*, we included interview date, age, sex, self-reported race and ethnicity (Hispanic, non-Hispanic Black, non-Hispanic White, and people of other races including American Indian, Alaskan Native, Asian, and Pacific Islander; race and ethnicity were analyzed in this study because there are known differences in outcomes by race and ethnicity, and systemic disinvestment in communities has resulted in differential exposure by these same characteristics), educational attainment (less than high school, general equivalency diploma, high school graduate, some college, or college or higher), ownership of primary residence, penalized splines of total household wealth with 5 *df*, urbanicity levels, neighborhood social economic status derived from 11 US Census variables,^33^ and a set of spatial basis functions, which are thin-plate regression splines based on residential addresses. We selected 10 *df* for these spatial basis functions on the basis of the Akaike information criterion of our fitted model following the instruction of Keller and Szpiro.^12^ We also included smoking status (never, current, or former), drinking status (no, low to moderate, or heavy consumption), and body mass index (calculated as weight in kilograms divided by height in meters squared) as potential mediator-outcome confounders. Next, we estimated the association of PM_2.5_ with the mediators with a logistic regression model as shown in Equation 2:

logit[*P*(stroke* = *1|PM_2.5_, *C*)] = β_0_ + β_1_PM_2.5_ + β′*C *(2),

where the *C* is the same matrix included in Equation 1. Finally, we used the regression parameters from the 2 models to define the CDA, INTref, INTmed, PIA, and estimated the proportions of the total excess association due to each component following the derivations given by VanderWeele.^31^ We reported associations scaled to an IQR difference and generated 95% CIs for each association using nonparametric bootstrap.

We conducted various sensitivity analyses to test the robustness of our findings. First, given that we previously observed larger associations between PM_2.5_ and dementia among younger participants,^26^ we conducted our mediation analyses stratified by age at baseline. We also examined our results restricted to time periods with higher quality PM_2.5_ estimates (ie, 2006 and after). Finally, to ensure the temporality between the exposure and mediator, we reran our analyses using incident stroke as the mediator (excluding participants with a history of stroke at baseline). Although we did not conduct this sensitivity analysis for hypertension due to the high prevalence at baseline, we evaluated prevalent stroke as a mediator among our participants stratified by their self-reported hypertension status at baseline. We performed our data analyses from August to November 2022 using R statistical software version 4.2.1 (R Project for Statistical Computing) and SAS statistical software version 9.4 (SAS Institute). The code to implement the causal mediation method and estimate the 95% CIs for this approach was taken from the appendices of VanderWeele,^31^ Discacciati et al,^34^ and Valeri et al.^35^

## Results

Our study included 27 857 participants (mean [SD] age at baseline, 61 [10] years; 15 747 female participants [56.5%]; 19 249 non-Hispanic White participants [69.1%]; mean [SD] follow-up time 10.2 [5.6] years) of whom 4105 (14.7%) developed dementia and 23 752 (85.3%) did not develop dementia (Table 1). The IQR of baseline PM_2.5_ concentrations was 10.9 to 14.9 μg/m^3^. Among participants with dementia, 2204 (53.7%) had a history of hypertension at baseline and 386 (9.4%) received a diagnosis of hypertension during the follow up. A total of 378 participants (9.2%) had a history of stroke at baseline and 673 (16.4%) had a stroke over the follow-up period. Compared with those without dementia, participants with incident dementia were older, more likely to have a history of hypertension and stroke at baseline, had a higher incidence of stroke during follow-up, and had higher levels of exposure to PM_2.5_. However, they had a lower incidence of hypertension during follow-up compared with those without dementia.

**Table 1.** Descriptive Statistics for Baseline Characteristics, Potential Mediators, and PM_2.5_ Concentrations Stratified by Incident Dementia During Follow-Up From 1998 to 2016 in the Health and Retirement Study

Characteristic	Participants, No. (%)		
	All (N = 27 857)	Dementia-free over follow-up (n = 23 752)	Incident dementia during follow-up (n = 4105)
Follow-up, mean (SD) y	10.2 (5.6)	10.5 (5.6)	8.3 (5.1)
Age, mean (SD), y	61 (10)	60 (9)	68 (10)
Sex			
Male	12 110 (43.5)	10 516 (44.3)	1594 (38.8)
Female	15 747 (56.5)	13 236 (55.7)	2511 (61.2)
Race and ethnicity			
Hispanic	3164 (11.4)	2589 (10.9)	575 (14.0)
Non-Hispanic Black	4654 (16.7)	3756 (15.8)	898 (21.9)
Non-Hispanic White	19 249 (69.1)	16 710 (70.4)	2539 (61.9)
People of other races^a^	790 (2.8)	697 (2.9)	93 (2.3)
Educational attainment			
Less than high school	5825 (20.9)	4045 (17.0)	1780 (43.4)
General education development	1369 (4.9)	1175 (4.9)	194 (4.7)
High school	8278 (29.7)	7144 (30.1)	1134 (27.6)
Some college	6561 (23.6)	5972 (25.1)	589 (14.3)
College and above	5824 (20.9)	5416 (22.8)	408 (9.9)
Own primary residence	21 572 (77.4)	18 649 (78.5)	2923 (71.2)
Baseline net worth without primary residence, mean (SD) $	242 012 (1 010 704)	256 381 (1 074 788)	158 873 (490 124)
Neighborhood social economic status, mean (SD) score	0.23 (0.92)	0.20 (0.93)	0.40 (0.88)
Urbanicity			
Urban	14 475 (52.0)	12 503 (52.6)	1972 (48.0)
Suburban	6028 (21.6)	5108 (21.5)	920 (22.4)
Ex-urban	6201 (22.3)	5218 (22.0)	983 (23.9)
Mediator status			
Stroke at baseline	1337 (4.8)	926 (3.9)	378 (9.2)
Stroke during follow-up	2284 (8.2)	1567 (6.6)	673 (16.4)
Hypertension at baseline	13 037 (46.8)	10 759 (45.3)	2204 (53.7)
Hypertension during follow-up	3844 (13.8)	3491 (14.7)	386 (9.4)
10-y PM_2.5_ exposure, mean (SD) μg/m^3^	12.96 (3.22)	12.8 (3.20)	13.8 (3.14)

^a^ People of other races included American Indian, Alaskan Native, Asian, and Pacific Islander.

**Figure 3.**
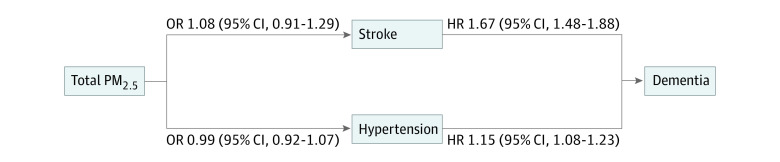
Illustration of the Association of PM_2.5_ Exposure With Dementia and Potential Mediators Odds ratios (ORs) and 95% CIs were used to analyze the association of PM_2.5_ exposure with potential mediators (left side) using logistic regression, and hazard ratios (HRs) and 95% CIs were used to analyze the association of potential mediators with dementia (right side) using proportional hazard regression. The models were assessing the association of an IQR difference (10.9-14.9 μg/m^3^) in PM_2.5_ with vascular conditions and were adjusted for age, interview date, sex, race and ethnicity, educational attainment, ownership of the primary residence, quartile of the total household wealth, urbanicity levels, neighborhood social economic status, and spatial basis functions (10 *df*), whereas the models assessing the association of vascular conditions with dementia were also adjusted for PM_2.5_.

After adjustment for all covariates, we found that higher levels of PM_2.5_ were not associated with increased risk of stroke (odds ratio [OR] per IQR difference in PM_2.5_, 1.08; 95% CI, 0.91-1.29) (Figure 3). There were associations of prevalent stroke and hypertension with incident dementia. The HRs of dementia were 1.67 (95% CI, 1.48-1.88) for those with prevalent stroke at baseline and 1.15 (95% CI, 1.08-1.23) for those with prevalent hypertension at baseline. There was no association of PM_2.5_ with prevalent hypertension (OR per IQR increment in PM_2.5_, 0.99; 95% CI, 0.92-1.07).

Table 2 shows the results of our formal causal mediation analysis. The adjusted HR for the total association of PM_2.5_ with incident dementia was 1.04 (95% CI, 0.98-1.11) per IQR. When we decomposed this total association into its 4 components using prevalent stroke as a putative mediator, there was no evidence that an interaction of PM_2.5_ with stroke existed through direct (INTref) or indirect (INTmed) paths, and virtually all of the association was direct. For prevalent hypertension, we found no evidence of mediation; however, we observed an interaction of the direct association with INTref accounting for 39.2% (95% CI, −138.5% to 216.9%) of the total excess association (1.04 – 1 = 0.04; 95% CI, −0.02 to 0.11), although the 95% CIs crossed 1, rendering the findings not statistically significant.

**Table 2.** Decomposition of the Association of PM_2.5_ With Incident Dementia Including Mediation and Interaction Associations by Prevalent Hypertension and Stroke Using Causal Mediation Analysis Among Health and Retirement Study Participants, 1998-2016

Association component	Hypertension		Stroke	
	HR (95% CI)	Percentage of excess association (95% CI)	HR (95% CI)	Percentage of excess Association (95% CI)
Total	1.03 (0.97 to 1.11)	100	1.04 (0.98 to 1.11)	100
Controlled direct association	1.02 (0.94 to 1.11)	61.9 (−113.8 to 237.6)	1.04 (0.98 to 1.11)	104.6 (91.0 to 118.2)
Reference interaction^a^	0.01 (−0.03 to 0.05)	39.2 (−138.5 to 216.9)	0.00 (−0.01 to 0.00)	−7.2 (−21.4 to 7.1)
Mediated interaction^a^	0.00 (0.00 to 0.00)	−0.2 (−2.1 to 1.6)	0.00 (0.00 to 0.00)	−0.5 (−2.1 to 1.0)
Pure indirect association	1.00 (1.00 to 1.00)	−0.8 (−6.6 to 5.0)	1.00 (1.00 to 1.00)	3.1 (−5.2 to 11.4)

Abbreviation: HR, hazard ratio.

^a^ Reference interaction and mediated interaction are the estimation of additive excess relative risk due to interaction using HRs.

In secondary analyses, we found similar results for PM_2.5_ from open fires as with the total PM_2.5_ (eTable 1 in Supplement 1), whereas we found that the association of PM_2.5_ from agriculture with incident dementia was attributable to the CDA only (eTable 2 in Supplement 1). Notably, since the total excess association was close to 0, the statistical properties for the proportion measures were unstable, so it may be inappropriate to interpret these measures.^31^ In our sensitivity analyses, we found consistent results when we limited our study to younger participants and time periods with higher quality exposure data; however, the INTref components through hypertension were not found in these populations (eTable 3 and eTable 4 in Supplement 1). Using incident stroke as the mediator also did not change our findings (eTable 5 in Supplement 1). Similarly, we found no evidence of any mediation or interaction through prevalent stroke among those with and without hypertension. However, we only observed the total association of PM_2.5_ with dementia among those with hypertension at baseline and not those without hypertension at baseline, offering further evidence of a potential interaction between PM_2.5_ and hypertension in relation to dementia incidence (eTable 6 in Supplement 1).

## Discussion

In this nationally representative cohort study in the US, we found no evidence to support stroke or hypertension as important mediators of the association of air pollution with incident dementia. Although the evidence suggesting that the burden from PM_2.5_ was greater among those with hypertension through a direct interaction did not meet statistical significance, our sensitivity analysis also suggested that there was only an association of PM_2.5_ with incident dementia among those with hypertension at baseline. No similar interaction was found for stroke. To our knowledge, this study is the first to use causal mediation modeling to quantify the degree to which 2 important vascular conditions could be mediators and/or modifiers of the association of PM_2.5_ with incident dementia.

At least 2 previous studies^6,22^ have evaluated a vascular mechanistic pathway of PM_2.5_ on dementia using formal mediation analysis approaches without evaluating association modification. In 1 study of 34 491 older residents in Canada,^22^ between 5% and 20% of the total association (HR, 1.03; 95% CI, 1.00-1.05 per 1 μg/m^3^) of PM_2.5_ was found to be explained by incident CVD events. A notable difference between that study^22^ and our work is the definitions of vascular conditions. Although those studies^6,22^ included many types of heart disease (eg, coronary heart disease, arrhythmia, or congestive heart failure), we only considered stroke and hypertension as our mediators. As a result, we might not have found a mediating role of vascular conditions if the key pathways between PM_2.5_ and dementia operated through heart diseases unrelated to hypertension or stroke. Notably, however, the second study of 2927 participants in Sweden^6^ attributed 49% of the total association of PM_2.5_ with dementia (OR, 1.89; 95% CI, 1.13-3.17 per 1 μg/m^3^) to an indirect pathway through baseline stroke.

Other studies have attempted to investigate the importance of vascular mechanisms using informal mediation approaches. For example, a study^36^ of 12 million Medicare beneficiaries (aged ≥65 years) in the US evaluated associations of PM_2.5_ with dementia, adjusting for hypertension, stroke, and heart failure. Consistent with our findings, they did not find evidence that their observed associations were reduced by these adjustments. Similar findings were seen in 2 other studies^10,12^ in the US with simple adjustments for CVD. Collectively, this literature indicates that the findings remain mixed regarding the importance of vascular conditions as a mediator of the association of PM_2.5_ with dementia.

Our lack of evidence for effect measure modification by hypertension and stroke is generally consistent with previous findings. In the aforementioned Swedish study,^6^ higher risk of dementia associated with PM_2.5_ was observed only in persons with heart failure (a downstream consequence of hypertension) but not those with atrial fibrillation, ischemic heart disease, or stroke. Also, in a study^37^ of a Hong Kong Chinese cohort of 66 830 older population as well as a study^38^ of a US cohort of 2239 older women, researchers found no solid evidence for effect measure modification by CVDs and its associated factors when using interaction terms in their models.

Although we hypothesized that hypertension and stroke would mediate or modify associations of PM_2.5_ with dementia, it is plausible that the association of air pollution with cognition may be mediated through other pathways. For example, particles are known to reach the brain through the olfactory, trigeminal, and vagus nerves and through peripheral circulation when breaching the blood-brain barrier, leading to local inflammation and oxidative stress.^39,40,41^ With observed associations of dementia in this cohort largely with PM_2.5_ from agriculture,^26^ it may be that these direct pathways are most important due to neurotoxic compounds in pesticides entering the brain.^42,43,44^ Additionally, although the evidence is not conclusive, recent studies^45,46,47,48,49^ suggest that there are mediated pathways through mental health disorders (eg, depression), poor sleep quality, and insulin resistance or disruption of the normal action of insulin in the brain.

### Strengths and Limitations

To our knowledge, this study is the first to formally evaluate the role of vascular conditions in the association of air pollution with incident dementia in the US using causal mediation models. By using a 4-way decomposition method, we were able to simultaneously examine the role of stroke and hypertension as modifiers and mediators. This work was conducted within a well-characterized, nationally representative cohort with PM_2.5_ concentrations estimated at participants’ residential addresses and dementia classified using a standardized approach from survey respondents and their proxies.

Despite these advantages, our study has some limitations. First, in contrast with our previous research that allowed for time-varying exposures,^26^ we used the 10-year average exposure before baseline because of the limited mediation methods for time-varying exposure in the survival setting. Sensitivity analyses in our past work in EPOCH indicate that this likely resulted in a 50% underestimation of the total association of PM_2.5_ with dementia,^26^ which may have reduced our power to capture mediation associations. Our estimation of PM_2.5_ levels on the basis of residential histories rather than real-time locations was another source of measurement error. However, since the mean age of our participants was 61 years and all were older than 50 years, they were less likely to leave their homes for work than were younger individuals. Similarly, our use of prevalent rather than incident hypertension and stroke may have introduced exposure measurement error given that we do not know whether the 10 years prior to baseline accurately captures the critical exposure window for affecting the intermediates. It may also be that associations with incident cases are larger than prevalent cases, although our sensitivity analysis using incident stroke indicated the robustness of our findings. Also, our binary indicator during follow-up may not have captured nuances of short-term blood pressure extremes, which have been known to be associated with dementia risk.^50^ In addition, our use of self-reported mediators may have introduced misclassification of hypertension and stroke. After adjustment for individual and neighborhood socioeconomic status and location, we expected that this error would be nondifferential and result in underestimation of the indirect effect and overestimation of the direct effect.^51^ Compared with stroke, misclassification of hypertension could be more likely, because asymptomatic undiagnosed hypertension is common, and some who were previously diagnosed may have reported no hypertension if their blood pressure was under control. We do not anticipate this being a large issue, however, given the high sensitivity and specificity of self-reported hypertension against measured blood measures in this cohort.^52^

## Conclusions

Contrary to our hypotheses, we did not find evidence that hypertension or stroke acted as mediators or modifiers of the association of PM_2.5_ with incident dementia. As a result, additional investigation of the pathways underlying the association of air pollution with dementia is needed to understand disease etiology and identify populations who might benefit most from pollution reduction strategies.

## Notes

Supplement 1. **eFigure.** Schematic Flowchart of the Study Population Selection **eTable 1.** The Association of PM_2.5 _From Open Fire With Incident Dementia Due to Mediation and Interaction With Prevalent Hypertension and Stroke Using Causal Mediation Analysis Among HRS Participants, 1998-2016 **eTable 2**. The Association of PM_2.5 _From Agriculture With Incident Dementia Due to Mediation and Interaction With Prevalent Hypertension and Stroke Using Causal Mediation Analysis Among HRS Participants, 1998-2016 **eTable 3.** The Association of PM_2.5 _With Incident Dementia Due to Mediation and Interaction With Prevalent Hypertension and Stroke Using Causal Mediation Analysis Among HRS Participants Younger Than 75 Years, 1998-2016 **eTable 4.** The Association of PM_2.5 _With Incident Dementia Due to Mediation and Interaction With Prevalent Hypertension and Stroke Using Causal Mediation Analysis Among HRS Participants Using an Alternative Time Period of 2006-2016 **eTable 5.** The Association of PM_2.5 _With Incident Dementia Due to Mediation and Interaction With Incident Stroke Using Causal Mediation Analysis Among HRS Participants, 1998-2016 **eTable 6.** The Association of PM_2.5 _With Incident Dementia Due to Mediation and Interaction With Prevalent Stroke Using Causal Mediation Analysis Among HRS Participants Stratified by Baseline Hypertension Status, 1998-2016

Supplement 2.
Data Sharing Statement
