# Supplementary material for: Hypertension and Stroke as Mediators of Air Pollution Exposure and Incident Dementia
Source: JAMA Netw Open. 2023 Sep 20;6(9):e2333470. doi: 10.1001/jamanetworkopen.2023.33470 (PMC10512106; doi:10.1001/jamanetworkopen.2023.33470)
Supplement: Supplement 1. — eFigure. Schematic Flowchart of the Study Population Selection eTable 1. The Association of PM2.5 From Open Fire With Incident Dementia Due to Mediation and Interaction With Prevalent Hypertension and Stroke Using Causal Mediation Analysis Among HRS Participants, 1998-2016 eTable 2. The Association of PM2.5 From Agriculture With Incident Dementia Due to Mediation and Interaction With Prevalent Hypertension and Stroke Using Causal Mediation Analysis Among HRS Participants, 1998-2016 eTable 3. The Association of PM2.5 With Incident Dementia Due to Mediation and Interaction With Prevalent Hypertension and Stroke Using Causal Mediation Analysis Among HRS Participants Younger Than 75 Years, 1998-2016 eTable 4. The Association of PM2.5 With Incident Dementia Due to Mediation and Interaction With Prevalent Hypertension and Stroke Using Causal Mediation Analysis Among HRS Participants Using an Alternative Time Period of 2006-2016 eTable 5. The Association of PM2.5 With Incident Dementia Due to Mediation and Interaction With Incident Stroke Using Causal Mediation Analysis Among HRS Participants, 1998-2016 eTable 6. The Association of PM2.5 With Incident Dementia Due to Mediation and Interaction With Prevalent Stroke Using Causal Mediation Analysis Among HRS Participants Stratified by Baseline Hypertension Status, 1998-2016 [file jamanetwopen-e2333470-s001.pdf]

Supplemental Online Content

Zhang B, Langa KM, Weuve J, et al. Hypertension and stroke as mediators of air pollution exposure and incident dementia. *JAMA Netw Open*. 2023;6(9):e2333470. doi:10.1001/jamanetworkopen.2023.33470

eFigure. Schematic Flowchart of the Study Population Selection

eTable 1. The Association of $PM_{2.5}$ From Open Fire With Incident Dementia Due to Mediation and Interaction With Prevalent Hypertension and Stroke Using Causal Mediation Analysis Among HRS Participants, 1998-2016

eTable 2. The Association of $PM_{2.5}$ From Agriculture With Incident Dementia Due to Mediation and Interaction With Prevalent Hypertension and Stroke Using Causal Mediation Analysis Among HRS Participants, 1998-2016

eTable 3. The Association of $PM_{2.5}$ With Incident Dementia Due to Mediation and Interaction With Prevalent Hypertension and Stroke Using Causal Mediation Analysis Among HRS Participants Younger Than 75 Years, 1998-2016

eTable 4. The Association of $PM_{2.5}$ With Incident Dementia Due to Mediation and Interaction With Prevalent Hypertension and Stroke Using Causal Mediation Analysis Among HRS Participants Using an Alternative Time Period of 2006-2016

eTable 5. The Association of $PM_{2.5}$ With Incident Dementia Due to Mediation and Interaction With Incident Stroke Using Causal Mediation Analysis Among HRS Participants, 1998-2016

eTable 6. The Association of $PM_{2.5}$ With Incident Dementia Due to Mediation and Interaction With Prevalent Stroke Using Causal Mediation Analysis Among HRS Participants Stratified by Baseline Hypertension Status, 1998-2016

This supplemental material has been provided by the authors to give readers additional information about their work.

© 2023 Zhang B et al. *JAMA Network Open.*

eFigure. Schematic Flowchart of The Study Population Selection.

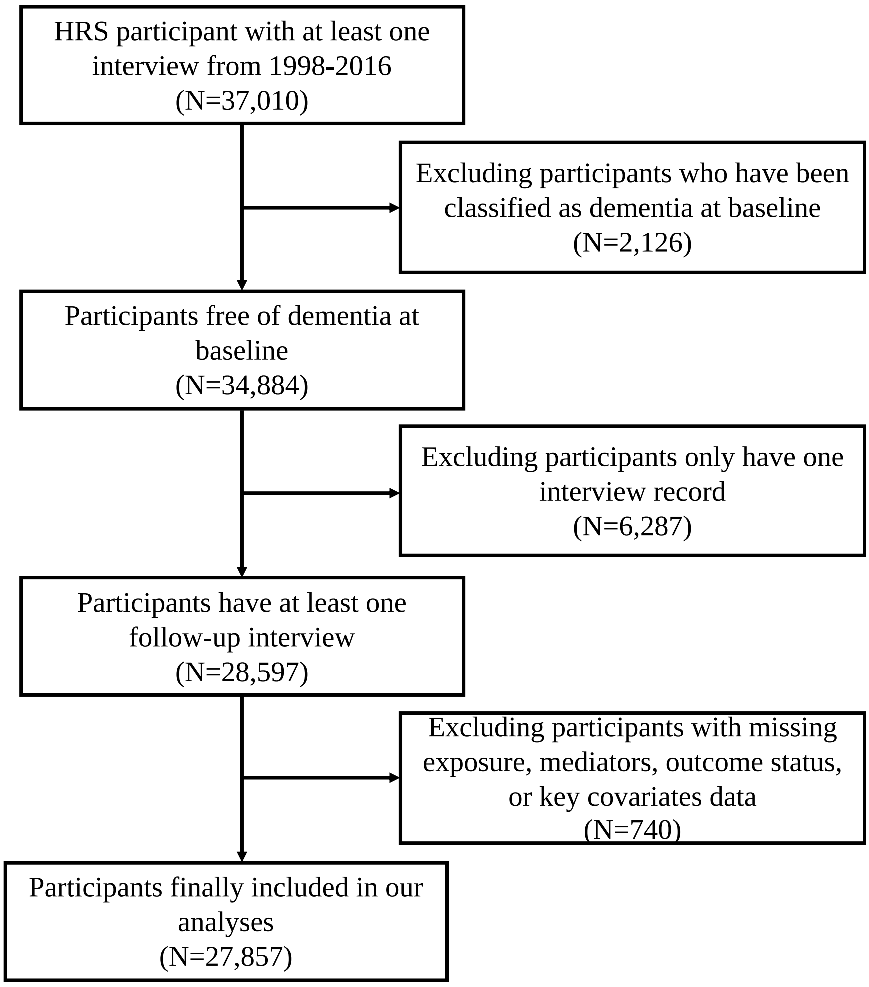

© 2023 Zhang B et al. *JAMA Network Open.*

eTable 1. The Association of $PM_{2.5}$ From Open Fire With Incident Dementia Due to Mediation and Interaction with Prevalent Hypertension and Stroke Using Causal Mediation Analysis Among HRS Participants, 1998-2016.

	**Hypertension**		**Stoke**	
	**HR**	**Percentage of Excess Association (%)**	**HR**	**Percentage of Excess Association (%)**
**Controlled Direct Association**	1.01 (0.99, 1.04)	53.6 (-25.9, 133.2)	1.02 (1.00, 1.05)	96.3 (87.5, 105.2)
**Reference Interaction[a]**	0.01 (-0.01, 0.03)	47.2 (-32.6, 126.9)	0.00 (-0.00, 0.00)	0.6 (-5.1, 6.3)
**Mediated Interaction[a]**	-0.00 (-0.00, 0.00)	-0.2 (-0.9, 0.5)	0.00 (-0.00, 0.00)	0.0 (-0.3, 0.3)
**Pure Indirect Association**	1.00 (1.00, 1.00)	-0.6 (-3.1, 1.9)	1.00 (1.00, 1.00)	3.0 (-2.3, 8.4)
**Total**	1.02 (1.00, 1.04)	100	1.02 (1.00, 1.05)	100

[a] Reference Interaction and Mediated Interaction is the estimation of additive relative excess risk due to interaction (RERI) using HR.

eTable 2. The Association of $PM_{2.5}$ From Agriculture With Incident Dementia Due to Mediation and Interaction with Prevalent Hypertension and Stroke Using Causal Mediation Analysis Among HRS Participants, 1998-2016.

	**Hypertension**		**Stoke**	
	**HR**	**Percentage of Excess Association (%)**	**HR**	**Percentage of Excess Association (%)**
**Controlled Direct Association**	1.07 (0.95, 1.23)	119.1 (30.4, 207.7)	1.09 (0.97, 1.24)	111.8 (95.2, 128.4)
**Reference Interaction[a]**	-0.01 (-0.06, 0.04)	-19.7 (-108.2, 68.9)	-0.01 (-0.01, -0.00)	-10.6 (-24.5, 3.3)
**Mediated Interaction[a]**	0.00 (-0.00, 0.00)	-0.2 (-1.8, 1.5)	0.00 (-0.00, 0.00)	0.8 (-2.1, 3.8)
**Pure Indirect Association**	1.00 (1.00, 1.01)	0.8 (-6.8, 8.3)	1.00 (0.99, 1.00)	-2.0 (-9.4, 5.3)
**Total**	1.07 (0.95, 1.21)	100	1.08 (0.97, 1.23)	100

[a] Reference Interaction and Mediated Interaction is the estimation of additive relative excess risk due to interaction (RERI) using HR.

© 2023 Zhang B et al. *JAMA Network Open.*

eTable 3. The Association of $PM_{2.5}$ With Incident Dementia Due to Mediation and Interaction with Prevalent Hypertension and Stroke Using Causal Mediation Analysis Among HRS Participants Younger than 75 Years, 1998-2016.

	Hypertension		Stoke	
	**HR**	**Percentage of Excess Association (%)**	**HR**	**Percentage of Excess Association (%)**
**Controlled Direct Association**	1.03 (0.94, 1.13)	109.6 (-45.8, 265.0)	1.04 (0.97, 1.12)	102.3 (89.0, 115.6)
**Reference Interaction[a]**	-0.00 (-0.05, 0.04)	-9.2 (-164.5, 146.1)	-0.00 (-0.01, 0.00)	-7.0 (-20.6, 6.6)
**Mediated Interaction[a]**	0.00 (-0.00, 0.00)	0.0 (-0.4, 0.5)	-0.00 (-0.00, 0.00)	-1.0 (-3.6, 1.5)
**Pure Indirect Association**	1.00 (1.00, 1.00)	-0.4 (-10.1, 9.3)	1.00 (1.00, 1.01)	5.7 (-7.6, 19.0)
**Total**	1.03 (0.96, 1.11)	100	1.04 (0.97, 1.12)	100

[a] Reference Interaction and Mediated Interaction is the estimation of additive relative excess risk due to interaction (RERI) using HR.

eTable 4. The Association of $PM_{2.5}$ With Incident Dementia Due to Mediation and Interaction with Prevalent Hypertension and Stroke Using Causal Mediation Analysis Among HRS Participants Using An Alternative Time Period of 2006-2016.

	Hypertension		Stoke	
	**HR**	**Percentage of Excess Association (%)**	**HR**	**Percentage of Excess Association (%)**
**Controlled Direct Association**	1.06 (0.98, 1.16)	114.8 (37.9, 191.8)	1.06 (0.99, 1.15)	100.9 (92.2, 109.5)
**Reference Interaction[a]**	-0.01 (-0.05, 0.03)	-15.5 (-92.5, 61.5)	-0.00 (-0.01, 0.00)	-3.3 (-10.7, 4.0)
**Mediated Interaction[a]**	-0.00 (-0.00, 0.00)	-0.1 (-0.9, 0.7)	-0.00 (-0.00, 0.00)	-0.3 (-1.1, 0.5)
**Pure Indirect Association**	1.00 (1.00, 1.00)	0.8 (-2.7, 4.3)	1.00 (1.00, 1.01)	2.8 (-3.5, 9.0)
**Total**	1.06 (0.98, 1.15)	100	1.06 (0.99, 1.15)	100

[a] Reference Interaction and Mediated Interaction is the estimation of additive relative excess risk due to interaction (RERI) using HR.

© 2023 Zhang B et al. *JAMA Network Open.*

eTable 5. The Association of $PM_{2.5}$ With Incident Dementia Due to Mediation and Interaction with Incident Stroke Using Causal Mediation Analysis Among HRS Participants, 1998-2016.

	HR	Percentage of Excess Association (%)
**Controlled Direct Association**	1.08 (1.01, 1.16)	116.3 (100.8, 131.8)
**Reference Interaction**[a]	-0.01 (-0.02, -0.00)	-13.8 (-27.0, -0.6)
**Mediated Interaction**[a]	0.00 (-0.00, 0.00)	1.0 (-0.7, 2.6)
**Pure Indirect Association**	1.00 (1.00, 1.00)	-3.4 (-9.3, 2.4)
**Total**	1.07 (1.00, 1.15)	100

[a] Reference Interaction and Mediated Interaction is the estimation of additive relative excess risk due to interaction (RERI) using HR.

eTable 6. The Effect of $PM_{2.5}$ on Incident Dementia Due to Mediation and Interaction with Prevalent Stroke Using Causal Mediation Analysis Among HRS Participants Stratified by Baseline Hypertension Status, 1998-2016.

	Hypertension (n=13,037)		Non-Hypertension (n=14,820)	
	HR	Percentage of Excess Association (%)	HR	Percentage of Excess Association (%)
**Controlled Direct Association**	1.08 (1.00, 1.16)	105.4 (94.6, 116.2)	0.99 (0.89, 1.10)	87.1 (-83.3, 257.6)
**Reference Interaction**[a]	-0.01 (-0.01, 0.00)	-6.9 (-17.0, 3.1)	0.00 (-0.01, 0.00)	20.7 (-246.5, 287.9)
**Mediated Interaction**[a]	0.00 (0.00, 0.00)	-0.5 (-2.1, 1.1)	0.00 (0.00, 0.00)	2.5 (-30.4, 35.4)
**Pure Indirect Association**	1.00 (1.00, 1.01)	2.0 (-4.3, 8.4)	1.00 (1.00, 1.00)	-10.4 (-145.6, 124.9)
**Total**	1.08 (1.00, 1.15)	100	0.99 (0.89, 1.10)	100

[a] Reference Interaction and Mediated Interaction is the estimation of additive relative excess risk due to interaction (RERI) using HR.

© 2023 Zhang B et al. *JAMA Network Open.*
