# Supplementary material for: Hypertension and Stroke as Mediators of Air Pollution Exposure and Incident Dementia
Source: JAMA Netw Open. 2023 Sep 20;6(9):e2333470. doi: 10.1001/jamanetworkopen.2023.33470 (PMC10512106; doi:10.1001/jamanetworkopen.2023.33470)
Supplement: Supplement 2. — Data Sharing Statement [file jamanetwopen-e2333470-s002.pdf]

Data Sharing Statement

Zhang. Hypertension and Stroke as Mediators of Air Pollution Exposure and Incident Dementia. *JAMA Netw Open*. Published September 20, 2023.
doi:10.1001/jamanetworkopen.2023.33470

Data
Data available: No
